# Comprehensive Care in Critical Services: A Spanish Qualitative Study

**DOI:** 10.3390/healthcare13070745

**Published:** 2025-03-27

**Authors:** Rocío de Diego-Cordero, Thalía Flores-Alpresa, Miriam Fernández-Rodríguez, Juan Vega-Escaño, José Miguel Pérez-Jiménez

**Affiliations:** 1 Department of Nursing, School of Nursing, Physiotherapy and Podiatry, University of Seville, 41009 Seville, Spain; rdediego2@us.es; 2 Research Group CTS1149: Comprehensive and Sustainable Health: Bio-Psycho-Social, Cultural and Spiritual Approach to Human Development, 41009 Seville, Spain; 3 San Hilario Health Center, Dos Hermanas, 41701 Seville, Spain; thaliafloresalpresa@gmail.com; 4 Juan Ramón Jiménez University Hospital, 21005 Huelva, Spain; miriammfr5@gmail.com; 5 Anesthesiology and Resuscitation Clinical Management Unit, University Hospital Virgen Macarena, 41009 Sevilla, Spain

**Keywords:** critical care, interviews, qualitative research, comprehensive healthcare, emergency service hospital

## Abstract

Background/Objectives: Comprehensive care is crucial in emergency healthcare. In intensive care units, a holistic approach may be difficult to implement due to the conditions of the patients and existing work protocols aimed at maintaining vital functions for the survival of patients. The present study aims to explore and describe the knowledge, attitudes, and perceptions of critical care and emergency health professionals regarding the implementation of integrated care practices within intensive care units, with the goal of identifying barriers and facilitators to adopting a holistic approach in emergency healthcare settings. Methods: This study implemented an exploratory and descriptive qualitative design with a phenomenological approach through semi-structured interviews with health professionals who had worked in intensive care units or emergency services for both public and private health institutions in Spain (n = 25). The study was conducted during the years 2023 and 2024, using a convenience sampling method along with snowball sampling, and a narrative discourse analysis was performed. The MAXQDA 2022 software program was used. This study was granted due permission by the Research Ethics Committee belonging to the Junta de Andalucía, under protocol code 0768-N-20. Results: The total sample consisted of 25 healthcare professionals from critical care and emergency services in Spain. The main themes, as key findings, were knowledge and perception, determining factors, resources and infrastructure, the bioethical dimension, perspectives on comprehensive care, and multidimensional impact. Most of the professionals were familiar with comprehensive care, but lack of resources and time prevented them from carrying it out in their units. Conclusions: For critical care and emergency professionals, comprehensive care is important to their clinical practice, but barriers to its realization still exist. Understanding the importance to these professionals of the application of comprehensive care is fundamental to establishing measures for its implementation in these services. It is also a motivation to continue providing humanized and compassionate care that respects the patient’s dignity. It is a priority to provide the necessary infrastructure and human resources so that patients admitted to these units can be cared for with this tool.

## 1. Introduction

Due to the global health crisis caused by the increasingly pressing shortage of human resources, in Spanish hospitals the number of hospitalizations in intensive care units (ICUs) has led to a considerable increase in the workload of nurses, whose numbers are well below the nurse–patient ratio worldwide [1]. According to the WHO, Spain is 61st in the world ranking, with a ratio of 5.73 vs. 8.77 nurses per 1000 inhabitants, while in terms of doctors we are in 26th position [2].

Intensive care and emergency units are environments characterized by work overload, state-of-the-art technology, the execution of complex tasks, stress, and the creation of defensive emotions during the care of highly complex patients. In the ICU, sometimes the work protocols and the conditions of patients make the holistic approach to care difficult [3].

In this environment, it is common to break the patient’s connection with his or her environment, which means that humanized care is depended on as the central axis [4].

Comprehensive healthcare refers to “the set of actions that promote and facilitate efficient, effective and timely care, which is directed more at people considered in their physical and mental integrity, as social beings belonging to different families and communities, who are in a permanent process of integration and adaptation to their physical, social and cultural environment, rather than at the patient or the disease as isolated facts” [5,6]. Therefore, in the units to which we refer, it becomes more necessary and is shown as a guarantee of high-quality care, taking into account not only the physical and symptomatic aspects of the patient, but also the entire global, biological, psychological, social, cultural, and spiritual sphere [7].

In most patients, being admitted to or visiting the emergency room of a hospital generates anxiety, motivated by fear of the unknown, of a doubtful future, and possible inconveniences of their illnesses, placing them in a moment of vulnerability and weakness in their lives [3]. Therefore, it is necessary to offer them comprehensive care, which guarantees an approach to all phases and dimensions of the person, together with multidisciplinary work [8].

A great emotional and spiritual confusion has been generated in patients, relatives, and professionals. Knowing and understanding these complexities allows the building of more coherent and humane interventions, which are necessary not only to provide an immediate response at the time of the disaster but also to build an effective, empathetic, and assertive accompaniment, taking into account the biopsychosocial sphere of patients [9].

Comprehensive care has been discussed in healthcare for years, and research in this area has been scarce. The technological progress achieved in the diagnosis and treatment of diseases has not gone hand in hand with progress in the development of nontechnical skills in healthcare teams (awareness, humanization, empathy, assertiveness, etc.). This is why patients and their families yearn for comprehensive care, even more so in the context of crisis and hospitalization in an ICU [7].

Previous studies have shown that there has been a deterioration in the quality of humanized care and that it is very necessary to know what aspects hinder the provision of humanized care in the act of caring, since the results will facilitate knowing the reality for the fundamental progress of patients and health professionals [10]. Still, research in the field of comprehensive care in emergency departments and emergency services continues to be insufficient. Furthermore, it is evident that there is a wide variety of interventions orientated towards integrated care in emergency departments; however, it is difficult to put these into practice due to the short time required in this area, as work in these departments leads to emotional overload and stress for the nursing professionals [11]. Interventions targeting psychological resilience are needed to reduce nurses’ stress perceptions [12].

The goal of this study was to explore and describe the knowledge, attitudes, and perceptions of critical care and emergency health professionals regarding the implementation of integrated care practices within intensive care units, with the goal of identifying barriers and facilitators to adopting a holistic approach in emergency healthcare settings.

## 2. Materials and Methods

### 2.1. Research Design

An exploratory and descriptive qualitative design with a phenomenological approach was used [13], employing semi-structured interviews which aimed to describe the meaning of an experience by identifying themes and subthemes born out of participant observation and discourse [14].

A qualitative approach grants researchers significant flexibility, allows for a deeper understanding of participants’ perspectives, facilitates the development of multiple interpretations of respondents’ viewpoints on a topic, and helps uncover concerns that survey-based methods alone may overlook.

This approach helps researchers comprehend the lives and necessities of others by helping to recognize and to set aside theoretical and ideological biases and is characterized by (a) a conceptual orientation provided by a research team, (b) a focus on a discrete community, (c) a focus on a problem within a specific context, (d) a limited number of participants, (e) the use of participants who may hold specific knowledge, and (f) the use of selected episodes of participant observation [15].

### 2.2. Setting and Participants

The study was conducted with healthcare professionals (physicians, emergency room technicians, nurses, and nursing assistants) working in intensive care and emergency departments at several hospitals and primary care centers in southern Spain. Specifically, the participants included professionals from the Virgen del Rocío University Hospital (Seville), the Juan Ramón Jiménez University Hospital (Huelva), the Virgen de Valme University Hospital (Seville), the University Hospital of Jerez (Cádiz), and support services from the Southern and Eastern Districts of Seville (Seville).

In particular, the participating hospitals have modern ICUs that offer comprehensive care for critically ill patients. These ICUs are equipped with cutting-edge technology and a multidisciplinary team that works together to provide the best possible care. Furthermore, the hospitals and primary care centers have emergency services that operate 24 h a day, treating both medical and surgical emergencies. These services are designed to ensure prompt and effective care, prioritizing patient comfort and safety.

### 2.3. Sampling and Eligibility Criteria

Convenience sampling and the snowball method were carried out, according to which participants are selected based on predetermined criteria. The main criterion was the familiarity of the participants with the phenomenon under investigation. The researchers looked for informants who had collectively encountered the phenomenon, even though they also differed in their characteristics and in their own personal experiences, to support the achievement of the aims [14]. Thus, informational messages were sent through social networks, WhatsApp work groups, and by email. Supervisors of emergency services and ICUs from the different hospitals, who acted as key agents, were also contacted. Participants who responded to the informational messages disseminated through the aforementioned electronic media or were invited to participate voluntarily through key informants were enrolled in the study after accepting and signing the informed consent form sent to them via email. This sample recruitment procedure guaranteed the anonymity of the informants until the time of the interviews by the researchers.

Finally, participants were included provided they were health professionals working in (ICUs) or emergency services for both public or private health institutions in Spain and treating critically ill patients. While the ICU health professionals worked in hospitals, emergency care was provided in hospitals, primary care, and outpatient emergency units. Health professionals who were working outside ICUs or emergency services, as well as those not caring for patients (i.e., academic or management level), were excluded.

### 2.4. Data Collection

The data collection was performed through semi-structured interviews. These were conducted in two moments by 2 researchers: from March 2023 to June 2023 and from September 2023 to December 2024, in the Spanish language, and lasted approximately 50 to 60 min. Data collection continued until data saturation was reached.

The interviews occurred individually at a time convenient for the participants, that is, respecting their preferences. Most interviews were conducted in person, so the AudioLab mobile app (version 1.3) was used to record and transcribe the information obtained. When distance was a barrier, interviews were conducted remotely via Zoom videoconferences. The same app was also used to ensure no information was lost.

In the case of face-to-face interviews, with prior agreement between the researcher and the informant, breaks in the work environment, specifically in the staff lounge, were used, accompanied by a cup of coffee, facilitating relaxed and familiar situations as much as possible. The objective was for the informants to feel comfortable responding to the series of questions posed by the researcher following a semi-structured interview script, which facilitated the smooth and orderly development of the content. Meanwhile, the online interviews were conducted at the informants’ quietest moments, usually when they were free at home, thus facilitating their participation. On both occasions, distractions in the environment were avoided, thus ensuring the absence of additional participants. Although, especially in the first case, this seemed practically impossible to achieve, it was quite easy, since the rest of the work environment largely respected the moment.

An interview script was used, and the question script was designed to encourage participants to tell their personal experiences, including feelings and emotions, and often focus on a particular experience or specific events (Table 1). Following our conceptual framework and adopting a phenomenological approach, our objective was to elucidate and interpret the significance of an encounter, frequently accomplished through the identification of fundamental subordinate and major themes. The result of a phenomenological investigation is a comprehensive depiction of these themes, encapsulating the fundamental essence of a “lived” experience [14].

### 2.5. Data Analysis

A phenomenological approach, which followed the Amadeo Giorgi theory [16], was used for data analysis. The goal of Giorgi’s method is to identify and express the meanings that participants experience through the phenomenon under investigation. Ultimately, using Amedeo Giorgi’s phenomenology as a methodological basis in the study of everyday experiences allows us to understand the unique perspective from which participants perceive the real world, and the typical categories of daily life help clarify the interpretation given to the motivations and actions. In addition, thematic analysis, as described by [17], was also used, following these steps: (1) familiarization with the data; (2) generation of categories; (3–5) search, review, and definition of themes; and (6) final report, which was prepared with the statements of the informants, indicated by participant letters, gender, and age, and transcription, literal reading, and theoretical manual categorization were performed. Initially, some categories were designed that, applying the characteristic circularity of qualitative research, were expanded with other emerging categories that appeared during the development of the interviews: “knowledge and perception”, “determining factors”, “resources and infrastructure”, “bioethical dimension”, “perspective on comprehensive care”, and “multidimensional impact”, the latter two being emerging categories. In addition, analysis and treatment of MAXQDA 2022 qualitative data were performed. MAXQDA is a software program designed for use in qualitative, quantitative, and mixed-methods research.

### 2.6. Trustworthiness

Trustworthiness refers to the extent to which research findings can be considered credible, transferable, confirmable, and dependable. In qualitative research, establishing trustworthiness requires specific strategies to ensure the credibility, transferability, confirmability, and dependability of the study’s findings [18]. To address these aspects, several criteria are often considered, including credibility, transferability, confirmability, and dependability. Here is a breakdown of what was done specifically to ensure each of these criteria: (1) Credibility: This criterion refers to the accuracy and authenticity of the data and findings. To ensure credibility, member checking was conducted, where participants were asked to review and validate the findings. Additionally, prolonged engagement with the data allowed for a deeper understanding, ensuring that interpretations were grounded in the participants’ perspectives. Triangulation of data sources was also used, comparing findings across different interviews and observations to increase the credibility of the results. (2) Transferability: This refers to the extent to which the findings can be applied in other contexts. To enhance transferability, thick description of the research context, participants, and processes was provided, allowing readers to determine whether the findings could be transferred to similar situations or settings. Detailed contextual information about the participants’ backgrounds and the setting was also shared to support the reader’s assessment of transferability. (3) Confirmability: This criterion focuses on ensuring that the findings are based on the data and not researcher bias or personal perspectives. To ensure confirmability, a clear audit trail was maintained, documenting the research process, the decisions made, and how conclusions were drawn from the data. Peer debriefing was also carried out, where colleagues reviewed the research process and findings, providing feedback and ensuring that the conclusions reflected the participants’ perspectives rather than the researchers’ interpretation. (4) Dependability: Dependability refers to the consistency and reliability of the research process over time. To establish dependability, an in-depth research process was documented and audit trails were kept, which outlined the methodology, and decisions made throughout the study. Code–recode procedures were applied, where data were coded at different stages and then recoded to ensure consistency in the coding process. Additionally, the research team conducted regular discussions to ensure consistency in data interpretation and decision-making.

The primary concerns regarding trustworthiness during the preparation phases were related to the reliability of the data collection method, the sampling strategy, and the selection of an appropriate unit of analysis. To address these aspects, the authors followed a checklist which helped them to reflect on trustworthiness [19].

In addition, this research followed the Consolidated Criteria for Reporting Qualitative Studies (COREQ) [20] This is an all-encompassing checklist that encompasses essential elements of study design that need to be documented. The criteria within this checklist can assist researchers in detailing vital aspects of the research team, study methods, study context, findings, analysis, and interpretations. Previous research has indicated that these checklists have enhanced the quality of reporting in various study types relevant to each checklist. These reporting guidelines are presented in Table A1.

### 2.7. Ethical Considerations

The guidelines of the Declaration of Helsinki were complied with for the performance of this study, and acceptance was obtained from the Ethics Committee of the Junta de Andalucía on 21 December 2020, with the internal code 0768-N-20.

All participants gave their written informed consent, and all data were anonymized to protect participant confidentiality.

## 3. Results

### 3.1. Participants

The final sample consisted of 25 health professionals from critical care and emergency/emergency services. All of the professionals worked in emergency and emergency services. Regarding nationality, 100% were Spanish (Table 2). Of the initial sample recruited, 15 participants declined to participate for work-related reasons, lack of time, or personal reasons.

**Figure 1:**
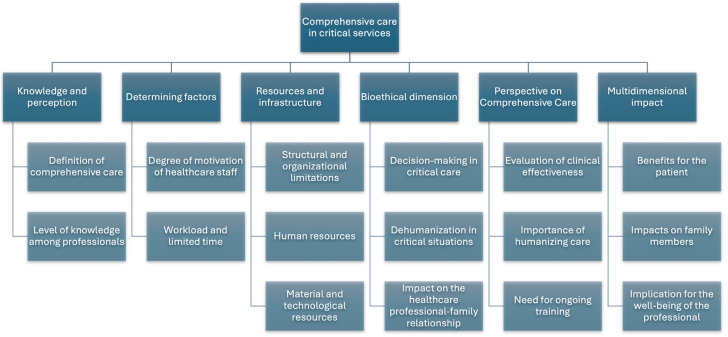
Coding tree with themes and subthemes.

Starting with the main theme of our study, the findings were systematically classified through an iterative process typical of qualitative research, resulting in six themes, two of which were emergent. Subsequently, these themes were refined and expanded with additional subcategories that emerged during the interviews. This classification of themes and subthemes is represented in the coding tree below and was informed by collaborative discussions among members of the research team (Figure 1).

### 3.2. Knowledge and Perception

First, the previous perceptions that the participants had about comprehensive care and the degree of training in this subject were investigated. Some professionals answered that they were unaware of the concept and needed a brief explanation of it. The rest described comprehensive care as a form of care and attention directed to the patient, where not only the person in question was considered but also their family, environment, and any event surrounding them.

“*In my opinion, the concept of comprehensive care is based on trying to provide the patient with care in all its aspects, that is, take into account both their family and their social, psychosocial aspect and obviously their physical health*”.

(p. 25)

“*If I tell you the truth, I don’t know what it. I could deduce it, and I could say that comprehensive care comes from integral, which means complete, so I would dare to say that comprehensive care is complete care, right? But… honestly it’s not a concept I know*”.

(p. 14)

### 3.3. Determining Factors

The professionals’ levels of motivation and the implications in relation to this subject were investigated. The majority provided motivating and demotivating considerations, but it is noteworthy that the last were more numerous. Generally, the interviewees were motivated by aspects such as offering quality care whose objective is well-being and progression to patient and family improvement, the opportunity to offer humanized assistance with empathy, the establishment of an assertive communication relationship between the patient and the professionals, and the sign of gratitude.

Regarding the reasons for demotivation, in the first place, we found that these derived from insufficient financing, high workloads, lack of time, and low professional–patient ratios caused by lack of staff. The lack of support from some health colleagues and the lack of empathy of the patients for the staff were also important.

“*What motivates me most are the benefits that this type of care brings the patient, for example, more confidence, since they are attended taking into account their needs*”.

(p. 23)

“*What motivates me to provide the most comprehensive care possible is the feedback that is generated with the patient, that response of comfort that translates into a climate of trust. In the end I think it is something that also challenges the professionals and the team, so more warmth is generated in the attention*”.

(p. 16)

### 3.4. Resources and Infrastructure

Regarding whether they considered the organization acceptable or insufficient, in terms of economic structure and human resources, to be able to offer comprehensive care, 96% of the participants stated that the primary resource required was economic. This was followed by personal resources, which were indicated by 88% of the participants; material resources, indicated by 76% of the participants; and training resources, indicated by 68% of the participants. In this regard, 11.7% of the participants highlighted the importance of emotional training. Additionally, 12% of those interviewed made reference to the importance of having an “introspective look” to detect the needs that should direct their healthcare.

“*To carry out comprehensive care, economic resources are needed above all, since having more money would make you have more staff, so there would be less workload and consequently a complete and quality care could be offered. In addition, this greater economic resource would also mean better health facilities, there would be more facilities and help for patients, in short, it would improve care*”.

(p. 19)

### 3.5. Bioethical Dimension

On this occasion, participants were asked whether they had experienced or observed any conflicts during their care work in relation to the comprehensive care of patients and whether they had experienced any ethical dilemmas in such circumstances. Ninety-six percent of the respondents answered affirmatively to both questions. The conflicts were related to situations of physical and mental exhaustion and isolation of patients, along with neglect of the family on many occasions, in addition to strict visiting rules. The professionals interviewed reported having experienced very difficult situations due to the peculiarity of the service, the high demand, and the scarcity of health resources.

“*The conflicts have been with the patients and above all with the relatives, because they were very afraid and did not know what they were facing, as most of them are sudden onset diseases. They were unable to understand that if we didn’t do more, it was because we couldn’t. Most of them demanded and shouted what they wanted. Most of them demanded and shouted what they wanted, even on the phone. You experience incredible anguish because they demand or reproach you for things that escape you, and your only objective is to seek a common good, which is not understood by many, you have no greater argument or explanation than that*”.

(p. 12)

“*We have experienced situations of intense tension. As a nurse transporting critical patients, I have experienced first-hand how the fate of several patients was decided, for example, who was transferred to the palliative care unit and who was transferred to a hospital to be treated as a palliative patient*”.

(p. 6)

### 3.6. Perspective on Comprehensive Care

Most of the professionals agreed that comprehensive care is an important element in healthcare, ensuring that it does not remain only in the physical sphere. It is an opportunity to offer individualized, complete, and humanized care, where the family takes a special interest, involving them in their loved one’s care.

“*I believe that comprehensive care is something important and useful for health, since it is a tool that gives you the opportunity to get to know your patient, to cover all their needs, to stop seeing them as a pathology to take them into account as the person who is, with his problems and his circumstances, aspects that directly influence his health, so that, by taking them into account and treating them, he contributes to his improvement*”.

(p. 1)

“*In an emergency unit, the fundamental thing is to solve the growing problem that threatens the patient’s life. However, we cannot forget everything that surrounds and influences that person, for example, his or her family or environment. These aspects can positively and negatively influence the patient’s evolution, which is why it is important to take them into account. This is achieved through comprehensive care*”.

(p. 22)

### 3.7. Multidimensional Impact

To conclude the interview, the health professionals were asked to assess comprehensive care as a healthcare provision tool and as a useful form of care for patients and their families and for the professionals themselves. One hundred percent of the interviewees answered in the affirmative, since they considered it a complete and beneficial form of care.

“*This type of care for family members is almost as important as for patients, or even more, because both have sensitive repercussions, I don’t know if I understand myself, what happens to the patient obviously affects him to the family; and the state of the family also ends with the state of the patient, so that, taking into account the holistic way to both, we will get more benefits*”.

(p. 8)

“*For me, as a professional, comprehensive care is beneficial because doing my job to the best of my ability is one of the most gratifying sensations I have ever experienced, which is why this type of care seems very important to me, because it is synonymous of quality and excellence, just what I want to offer my patients, as long as it is in my hands*”.

(p. 9)

## 4. Discussion

In this study, we learned about the knowledge, attitudes, and perceptions of critical care and emergency health professionals regarding comprehensive care. Insufficient funding, high workloads, lack of time, and low professional–patient ratios are the main reasons for the lack of motivation of these healthcare professionals to provide holistic care to patients. These aspects have already been identified in previous studies, in which it was noted that the lack of resources translates into a worsening of the conditions of healthcare services, which makes the situation more difficult and leads to a regression in the quality of care [11]. The healthcare professionals also pointed out that another demotivating element is the lack of empathy of patients and their families towards healthcare personnel. In this regard, previous studies have pointed out that human beings, by nature, tend to cooperate; however, when faced with a real or unreal situation of stress, fear, scarcity, or uncertainty, where they feel helpless, they become selfish, focusing only on their own needs without being aware of everything around them [21].

In our study, most of the healthcare staff interviewed believe that these are the causes that act as barriers to applying comprehensive, quality patient care, to which must be added the lack of training in these issues. The predominant opinion is, again, that more funds should be allocated to the recruitment of sufficient staff to be able to carry out such care. It should be noted that the whole situation is reflected in the occurrence of continuous episodes of ethical dilemmas that require psychological assistance. This measure is very important to avoid serious emotional pathologies in people affected by emergency situations [22].

Regarding the degree of knowledge that professionals have about the concept of comprehensive care, it is worth noting that a large proportion of the health professionals in this study offered more or less accurate definitions of it as a complete and quality form of care, with which all patient needs are covered [23].

Another of the objectives achieved was to learn about the attitude of these healthcare providers in the application of integrated care. In this sense, the professionals pointed out that they feel motivated to provide comprehensive care, in that it is the perfect way to offer complete and quality care, which provides the opportunity to humanize care, as reflected in the satisfaction of the patient and their family and in the results obtained. This point of view is supported by previous studies, which point out that the integrated care model is the most appropriate way to respond to the health needs of the individual, the family, and the community, thus achieving a collective good while providing quality healthcare [24].

In this research, the time factor became evident as a necessary resource for the comprehensive care of critical patients in urgent and/or emergency situations. The lack of time is linked, of course, to the lack of personnel—two resources that go hand in hand and which depend on a third one, the economic one. This point of view is reflected in Caldas’s article, which alludes to the need to put an end to cuts in healthcare, as they negatively affect the quality of care. Addressing comprehensive care does not require too much time, and key data may emerge that at first sight were not taken into account [21].

Most of the healthcare staff interviewed believe that it is possible to provide this type of integrated care in these services. The patient–professional ratios being unsustainable, they have increased the demands and impatience of patients and relatives and have exhausted the staff. The professionals are aware of the high cost involved because it requires a great effort, taking into account all the shortcomings of the system and the need for an adequate number of staff to maximize their capacities. This imbalance is not perceived by health professionals as a reason for discouragement to carry out and improve this care, however, as the return to “normality” implies the readoption of the forgotten comprehensive care [25].

There are various opinions about the emergence of ethical dilemmas regarding the relationship between comprehensive care/restrictions/critical patients. The participants have had to work through many of these conflicts, the most prominent being that of making decisions without taking into account the patient’s wishes. They point out that they sometimes felt that they dehumanized care [26].

Most professionals consider comprehensive care to be a very important element in healthcare, as they describe it as a useful means of getting to the bottom of certain problems that go beyond the physical sphere but that affect health and illness. Moreover, they add, it is the only way to create individualized, comprehensive, and humanized care, where the patient and the family are of the utmost importance. In this sense, it is a way for the family to participate in the care of their loved one, a way of involving them in the care process from which they felt marginalized, which positively affects the patient. In this regard, previous studies have pointed out that approaching the patient using integrated care is a way of treating them holistically and humanely [13].

Providing comprehensive care that meets all patients’ needs and involves their families increases their satisfaction and makes them feel useful and part of the process. The health professionals involved in this comprehensive process benefit from experiencing the patient’s gratitude and strengthening their professional identity, which motivates them to continue providing care that is humanized and respectful of the patient’s dignity. Integrated care is especially important for nurses, as it allows them to accompany and support patients in their health–illness process and to provide quality and timely care. It is a priority to establish measures that implement comprehensive care in emergency services. It is necessary to establish an emergency and urgency system whose main care is comprehensive and thus assists the population in a holistic manner. For comprehensive care to become a reality in these services, it is essential to allocate resources to make it possible, i.e., policies that regulate regulations and plan service networks [10].

### Limitations

This study presents certain limitations that should be considered. First, it was conducted in Spain and reflects the experiences of healthcare professionals working in Spanish medical facilities. As cultural backgrounds vary, it is challenging to ensure that the same results are observed in other countries. Second, a purposive sampling method was used, so generalizability should be approached with caution.

Future research should explore the perspectives and perceptions of emergency and critical care healthcare professionals across different cultural contexts, as societies may adopt distinct approaches to addressing comprehensive care. Another valuable avenue for future studies would be to compare how comprehensive care is addressed in other health contexts.

## 5. Conclusions

Integrated care is defined as a patient-centered approach that approaches individuals holistically, considering not only the presenting problem, but also the physical, psychological, and social aspects surrounding it. This model of care seeks to meet the needs of the patient in all their dimensions, promoting high-quality care. The primary motivation for health professionals in emergency and critical care situations to provide this type of care is the desire to provide care that responds to the patient’s needs and generates gratitude. However, increasing workloads and lack of recognition are demotivating factors.

Integrated care involves addressing all of the patient’s needs, not just medical needs, and focusses on physical, social, and mental well-being. Implementing this model requires financial resources, which are essential for hiring additional staff, developing training programs, and improving infrastructure. In many emergency health units, integrated care is not implemented due to the prioritization of technical functions that address acute health problems, relegating other aspects to the background.

Integrated care benefits the health of the patient and their family, promoting a sense of belonging and trust in the relationship with health professionals. However, the lack of time and staff can be a disincentive for professionals. Despite the limitations of the system, comprehensive care is essential and necessary, as patients require a humanized approach that transcends the health–illness paradigm.

Ethical dilemmas are common among staff, generated by stress, uncertainty, and physical and mental exhaustion. Comprehensive care is especially crucial at times such as the dignity–death process, as it allows the patient to feel understood and supported and family members to participate in the care, fostering a sense of usefulness. For health professionals, this approach reaffirms their professional identity and contributes to excellence in care. In the field of emergency care, comprehensive care is fundamental, as it represents the citizen’s first contact with health services and first responders, who are key in the detection of social and psychological emergencies.

## Figures and Tables

**Table 1:** Interview guide.

1. Are you familiar with the concept of integrated care? What does it mean to you?
2. In this comprehensive care, what are the reasons that motivate or discourage you to provide it?
3. What resources do you think are necessary to carry out comprehensive care (economic, material, personal, training…)?
4. In the situation you are living in due to the lack of human resources, do you think that this comprehensive care is still possible?
5. Have you had/observed any conflict during this health crisis in comprehensive care? Have you experienced any ethical dilemmas in these circumstances?
6. Finally, reflect on comprehensive care in the field of critical care and emergency care, how would you describe the level of importance, in what situations do you think this approach is most needed, do you think it would be helpful to your patients, to the patient’s family members, to yourself, and why?

**Table 2:** Participants’ characteristics.

Participant Code	Age (Years)	Gender	Work Experience (Years)	Current Position	Workplace	Service/ Department	Graduate Course
P1	25	Female	14	Nurse	Hospital	Adult hospital emergencies	Specialization in critical care, urgencies, and emergencies
P2	29	Male	8	Nurse	Hospital	Adult hospital emergencies	Specialization in critical care, urgencies, and emergencies
P3	28	Female	8	Physician	Hospital	Intensive care unit	Specialization in critical care, urgencies, and emergencies
P4	30	Female	10	Nurse	Hospital	Intensive care unit	Courses in critical care, urgencies, and emergencies
P5	31	Male	6	Nurse	Hospital	Adult hospital emergencies	Specialization in critical care, urgencies, and emergencies
P6	36	Male	8	Health emergency technician	Primary care	CCED/PCES *	Specialization in critical care, urgencies, and emergencies
P7	22	Female	8	Nurse	Primary care	CCED/PCES *	Specialization in critical care, urgencies, and emergencies
P8	26	Female	7	Nurse	Hospital	Intensive care unit	Specialization in critical care, urgencies, and emergencies
P9	58	Male	10	Health emergency technician	Primary care	CCED/PCES *	Courses in critical care, urgencies, and emergencies
P10	40	Male	6	Physician	Primary care	CCED/PCES *	Specialization in critical care, urgencies, and emergencies
P11	42	Male	6	Nurse	Hospital	Intensive care unit	Specialization in critical care, urgencies, and emergencies
P12	46	Female	9	Nurse	Hospital	Adult hospital emergencies	Specialization in critical care, urgencies, and emergencies
P13	38	Male	10	Nurse	Primary care	CCED/PCES *	Specialization in critical care, urgencies, and emergencies
P14	33	Female	7	Nurse	Hospital	Adult hospital emergencies	Courses in critical care, urgencies, and emergencies
P15	33	Male	8	Nurse	Primary care	CCED/PCES	Specialization in critical care, urgencies, and emergencies
P16	31	Female	11	Nurse	Hospital	Adult hospital emergencies	Specialization in critical care, urgencies, and emergencies
P17	34	Male	5	Nurse	Hospital	Gynecological emergencies	Specialization in critical care, urgencies, and emergencies
P18	32	Female	10	Nurse	Primary care	CCED/PCES *	Courses in critical care, urgencies, and emergencies
P19	44	Female	10	Technician in auxiliary nursing care	Hospital	Adult hospital emergencies	Specialization in critical care, urgencies, and emergencies
P20	29	Female	9	Nurse	Hospital	Adult hospital emergencies	Specialization in critical care, urgencies, and emergencies
P21	34	Male	8	Nurse	Primary care	CCED/PCES *	Courses in critical care, urgencies, and emergencies
P22	38	Female	5	Nurse	Primary care	CCED/PCES *	Specialization in critical care, urgencies, and emergencies
P23	37	Female	5	Nurse	Hospital	Gynecological emergencies	Specialization in critical care, urgencies, and emergencies
P24	25	Male	5	Nurse	Primary care	Transfers of critical patients	Courses in critical care, urgencies, and emergencies
P25	26	Male	5	Nurse	Primary care	Transfers of critical patients	Courses in critical care, urgencies, and emergencies

* CCED: Critical Care and Emergency Device; PCES: Primary Care Emergency Service.

## Data Availability

The original contributions presented in the study are included in the article, further inquiries can be directed to the corresponding authors.

## Appendix A

**Table A1:** Consolidated Criteria for Reporting Qualitative Studies (COREQ): 32-item checklist.

No.	Item	Guide Questions/Description	Response
	Domain 1: Research team and reflexivity		
Personal characteristics			
1.	Interviewer/facilitator	Which author/s conducted the interview or focus group?	All the interviews were conducted by two authors (Authors 1 and 5).
2.	Credentials	What were the researcher’s credentials (e.g., PhD, MD)?	Authors 1, 4, and 5 were PhD level. Authors 2 and 3 were MScN level.
3.	Occupation	What was their occupation at the time of the study?	Authors 1, 4, and 5 were research professors. Authors 2 and 3 worked as nurses.
4.	Gender	Was the researcher male or female?	Authors 1, 2, and 3 were females. Authors 4 and 5 were males.
5.	Experience and training	What experience or training did the researcher have?	All researchers had experience in carrying out qualitative research. Authors 1, 4, and 5 had training in social research. Authors 2 and 3 had been trained to conduct interviews.
Relationship with participants			
6.	Relationship established	Was a relationship established prior to study commencement?	No, there was not.
7.	Participant knowledge of the interviewer	What did the participants know about the researcher (e.g., personal goals, reasons for conducting the research)?	Name, occupation, reasons for conducting the research.
8.	Interviewer characteristics	What characteristics were reported about the interviewer/facilitator (e.g., biases, assumptions, reasons for exploring and interest in the research topic)?	Name, occupation, contact method, reasons for conducting the research.
Domain 2: Study design			
Theoretical framework			
9.	Methodological orientation and theory	What methodological orientation was stated to underpin the study (e.g., grounded theory, discourse analysis, ethnography, phenomenology, content analysis)?	Exploratory and descriptive qualitative design with a phenomenological approach through semi-structured interviews.
Participant selection			
10.	Sampling	How were participants selected (e.g., purposive, convenience, consecutive, snowball)?	Convenience sampling and snowballing.
11.	Method of approach	How were participants approached (e.g., face-to-face, telephone, mail, email)?	Telephone calls and video calls.
12.	Sample size	How many participants were included in the study?	25.
13.	Non-participation	How many people refused to participate or dropped out? Reasons?	15 for work-related reasons (mainly lack of time) and another for personal reasons.
Setting			
14.	Setting of data collection	Where was the data collected (e.g., home, clinic, workplace)?	The telephone calls and video calls were carried out by video-calling in different places.
15.	Presence of non-participants	Was anyone else present besides the participants and researchers?	No, there was not.
16.	Description of sample	What are the important characteristics of the sample (e.g., demographic data, date)?	Health professionals who had worked in intensive care units (ICUs) or emergency services for both public and private health institutions in Spain.
Data collection			
17.	Interview guide	Were questions, prompts, or guides provided by the authors? Was it pilot-tested?	Yes, they were./Yes, it was.
18.	Repeat interviews	Were repeat interviews carried out? If so, how many?	No, they were not.
19.	Audio/visual recording	Did the research use audio or visual recording to collect the data?	Audio and video recording.
20.	Field notes	Were field notes made during and/or after the interview or focus group?	Yes, they were (field notes).
21.	Duration	What was the duration of the interview or focus group?	Between 50 and 60 min.
22.	Data saturation	Was data saturation discussed?	Yes, it was.
23.	Transcripts returned	Were transcripts returned to participants for comment and/or correction?	No, it was not
Doman 3: Analysis and findings			
Data analysis			
24.	Number of data coders	How many data coders coded the data?	Two (Authors 2 and 3).
25.	Description of the coding tree	Did the authors provide a description of the coding tree?	Yes, we did.
26.	Derivation of themes	Were themes identified in advance or derived from the data?	Themes were derived using both methods.
27.	Software	What software, if applicable, was used to manage the data?	MAXQDA 2022
28.	Participant checking	Did participants provide feedback on the findings?	Reviewed by 2 informants.
Reporting			
29.	Quotations presented	Were participant quotations presented to illustrate the themes/findings? Was each quotation identified (e.g., participant number)?	Yes, there were./Yes, it was.
30.	Data and findings consistent	Was there consistency between the data presented and the findings?	Yes, there was.
31.	Clarity of major themes	Were major themes clearly presented in the findings?	Yes, they were.
32.	Clarity of minor themes	Is there a description of diverse cases or discussion of minor themes?	Yes, there is.

Source: [20].
